# Cardiac Troponin Elevation Predicts Mortality in Patients Undergoing Orthotopic Liver Transplantation

**DOI:** 10.1155/2013/252838

**Published:** 2013-07-14

**Authors:** David Snipelisky, Sean Donovan, Michael Levy, Raj Satyanarayana, Brian Shapiro

**Affiliations:** ^1^Department of Medicine, Mayo Clinic, Jacksonville, FL, USA; ^2^Division of Cardiovascular Diseases, Mayo Clinic, Jacksonville, FL, USA; ^3^Division of Gastroenterology and Hepatology, Mayo Clinic, Jacksonville, FL, USA

## Abstract

*Introduction*. While patients undergoing orthotopic liver transplantation (OLT) have high cardiovascular event rates, preoperative risk stratification may not necessarily predict those susceptible patients. Troponin T (TnT) may help predict patients at risk for cardiovascular complications. *Methods*. Consecutive patients undergoing OLT at Mayo Clinic in Florida between 1998 and 2010 who had TnT obtained within 10 days following surgery were included. Three groups were compared based on TnT level: (1) normal (TnT ≤0.01 ng/mL), (2) intermediate (TnT 0.02–0.11 ng/mL), and (3) elevated (TnT >0.11 ng/mL). Overall and cardiovascular mortality was assessed. *Results*. Of the 78 patients included, there was no difference in age, gender, severity of liver disease, and echocardiographic findings. Patients in the normal and intermediate TnT groups had a lower overall mortality rate (14.3% and 0%, resp.) when compared with those with elevated TnT (50%; *P* = 0.001). Patients in the elevated TnT group had a cardiovascular mortality rate of 37.5% compared with 1.4% in the other groups combined (*P* < 0.01). The elevated TnT group had a much higher mortality rate when compared with those in the intermediate group (*P* < 0.0001). *Conclusion*. TnT may accurately help risk stratify patients in the early postoperative setting to better predict cardiovascular complications.

## 1. Introduction

Despite a standardized and comprehensive preoperative cardiovascular evaluation, the peri- and postoperative cardiovascular event rate is high in patients with end-stage liver disease undergoing orthotopic liver transplantation (OLT) [1–3]. In fact, cardiovascular mortality remains the third leading cause of death following OLT and is responsible for 12%–16% of all deaths [3]. Conventional stress testing and risk-factor assessment may be inadequate to fully detect the burden of coronary artery disease in this population [1–6]. Due to the excessive postoperative cardiovascular event rate, an additional tool beyond careful clinical evaluation and noninvasive cardiovascular imaging to aid risk assessment may be of value. 

Cardiac troponin is a contractile protein located within the myocyte which may be detected by sensitive assays in the setting of myocardial necrosis [7]. Several studies have suggested that troponin is valuable in patients with both acute and chronic liver failure prior to transplantation. Such studies have shown that an elevated troponin preoperatively is indicative of worse peri- and postoperative outcomes, yet none have studied the relevance of troponin elevation post-transplantation [1–6]. Mechanisms of troponin elevation in these patients can occur in a variety of both cardiac and noncardiac related conditions, including sepsis and renal failure, which may deleteriously alter loading conditions onto the heart with or without the presence of myocardial ischemia. Additionally, patients undergoing liver transplantation have marked changes in sympathetic tone and cardiac output. This may lead to demand ischemia, which can also lead to early detection of troponin in the blood. Therefore, the overall significance of such an elevation is difficult to ascertain [4, 5, 7–10]. 

The aim of this study is to evaluate the prognostic significance of troponin T (TnT) elevation in patients undergoing OLT and to better understand cardiovascular mechanisms related to these changes. We believe that TnT may accurately predict mortality and aid in the perioperative assessment of these patients.

## 2. Methods

Consecutive patients who underwent OLT and had TnT levels obtained within 10 days following transplantation at Mayo Clinic in Florida from 1998 to 2010 were included. Clinical, demographic, echocardiographic, and noninvasive data were collected via chart review. In addition to type and severity of underlying liver disease, comprehensive assessment of risk factors for coronary artery disease was obtained. Obstructive coronary artery disease, defined as coronary artery stenosis >50% on invasive angiography, or prior myocardial infarction were assessed. Preoperative echocardiographic and stress perfusion imaging within one year prior to transplantation were included. Patients were classified as having a positive tobacco history with either a report of greater than five pack years of smoking history or tobacco use at date of transplantation. A creatinine of greater than 1.2 or a level twice the patient's baseline were used to classify patients as having renal dysfunction. The model for endstage liver disease (MELD) score was calculated on each patient based on the laboratory data set that correlates with the time of TnT blood draw. Thrombolysis in myocardial infarction (TIMI) and global registry of acute coronary events (GRACE) risk scores were calculated. Patients who were not deceased were excluded with less than 24 months of followup after transplantation. Mortality and cardiovascular events were obtained from chart review and social security death index database. The time to followup was determined by the date of the last clinical note, documented correspondence, or date of death for each patient. Each patient's data were only used once. If a patient had a subsequent liver transplantation, only data from the first transplantation was used. Institutional review board approval was obtained prior to the initiation of this study.

**Figure 1:**
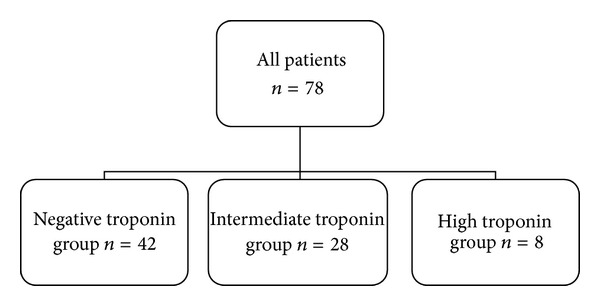
Stratification of patients within the study.

Patients were stratified according to TnT value into three groups: normal (≤0.01 ng/mL) TnT, intermediate (0.02–0.11 ng/mL) TnT, or elevated (>0.11 ng/mL) TnT (Figure 1) based on the single highest recorded value. All patients in the study had the same TnT assay performed. TnT groupings were based on normal TnT laboratory values at our institution.

Fisher's exact test and one-way analysis of variance (ANOVA) was used to analyze our data. *P* values less than 0.05 were considered to be significant. 

## 3. Results

Of 2010 patients who underwent OLT at our institution, 78 patients met the inclusion criteria and were included. There was no difference in baseline demographics between groups (Table 1). In the overall cohort, 15% had prior history of coronary artery disease although none had a prior myocardial infarction. Cardiovascular risk factors were commonly observed in the cohort, including diabetes (*n* = 25, 32%), systemic hypertension (*n* = 28, 36%), and prior history of smoking tobacco (*n* = 29, 37%). Over 70% of TnT values were obtained within a 3-day period of transplantation. All three groups had a similar time from transplant to TnT laboratory draw. Preoperative echocardiography was performed, and all patients had preserved left ventricular systolic function (mean ejection fraction of 65.1%). Three patients had evidence of right ventricular dysfunction and mildly elevated pulmonary pressures were commonly observed (RV mean pressure 27.8 mm Hg), and those with elevated pulmonary pressures were found to have higher overall left ventricular ejection fractions. The majority of patients (91%) underwent preoperative stress testing. None had findings suggestive of ischemia on noninvasive testing, and no patient underwent preoperative invasive coronary angiography. 

The main causes of cirrhosis included viral hepatitis (39%), nonalcoholic steatohepatitis (14%), and alcohol (13%). All patients but one underwent transplantation for chronic liver failure. One patient developed acute fulminant liver failure from acetaminophen toxicity (Table 2). There was a difference in the MELD scores between the three patient groups (*P* = 0.003), with the elevated TnT group having a MELD score of 14.0, the intermediate of 13.1, and the normal group with a score of 8.9 suggesting more advanced disease in the elevated TnT group (*P* = 0.011). The higher MELD score was predominantly affected by INR value (*P* = 0.011), although the creatinine level tended to increase among the three groups (*P* = 0.093) (Table 3). Creatinine level showed no significant differences between the high and intermediate groups (*P* = 0.889). 

Patients without TnT elevations were found to have a higher percentage (59.5%) of normal electrocardiogram results when compared with the intermediate (50.0%) and the elevated TnT (12.5%) groups, yet there was no statistical significance in this relationship. The elevated TnT group had a higher rate of new-onset right bundle branch blocks (12.5%) compared with the intermediate (0.0%) and negative (2.38%) groups. Further, the elevated TnT group had a higher rate of new-onset left bundle branch blocks (12.5%) when compared with the intermediate (0.0%) and negative (0.0%) groups (Table 3).

TIMI and GRACE risk scores did not show significant differences between the patients in the elevated TnT group. Patients that had died from a documented cardiac cause had an average TIMI score of 2.3 and GRACE of 110, compared to a TIMI of 2 and GRACE of 103.5 in those that were alive at followup.

**Figure 2:**
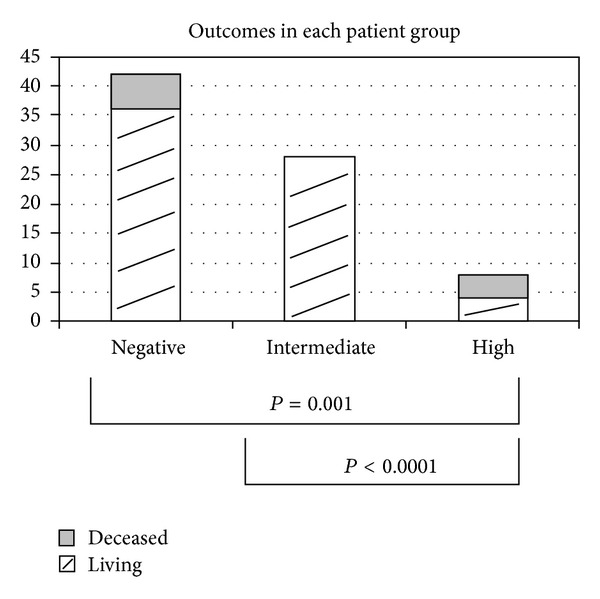
Overall outcomes in each patient group.

**Figure 3:**
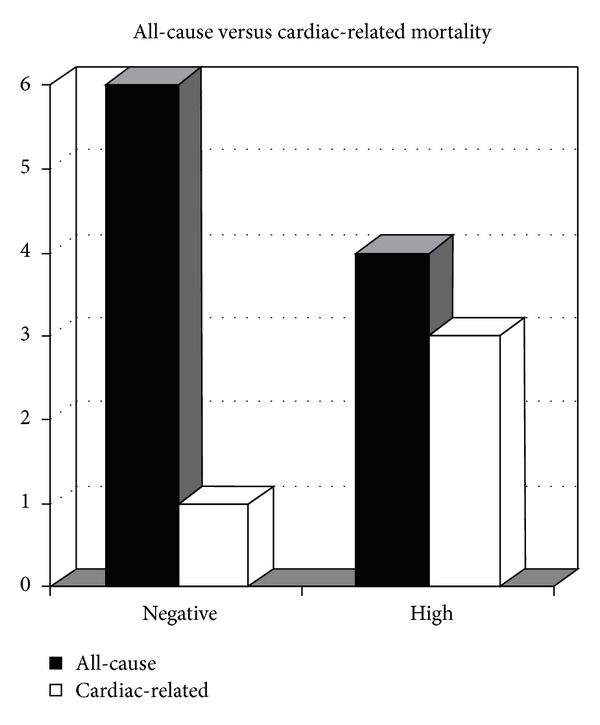
All-cause versus cardiac-related mortality in the negative versus high troponin groups. Note No deaths were observed in the intermediate troponin group.

Overall, 87.2% of all patients were alive at a followup period of at least two years. Eighty-six percent, 100%, and 50% of patients were alive in the negative, intermediate, and elevated TnT group, respectively (Figure 2). Out of those patients who had died, 16.7% (*n* = 1) were directly related to cardiac causes in the negative group, none in the intermediate group, and 75% in the elevated TnT group (*n* = 3) (Figure 3). Other causes of death were from liver rejection/failure, sepsis, and unknown causes classified as natural death in the medical record (Table 4). Comparisons between the elevated TnT and intermediate (*P* < 0.0001), negative (*P* = 0.04) and intermediate and negative combined (*P* < 0.01) groups all showed strong significant difference in overall mortality.

## 4. Discussion

 Our study found a significant difference between TnT levels and mortality between the negative, intermediate, and elevated TnT groups. Specifically, the elevated TnT group had a higher rate of both all-cause and cardiac mortality when compared with the negative and intermediate patient groups. Eighty-seven percent of patients were noted to be living with at least two years of followup, which is comparable to Mayo Clinic's three-year average survival rate of 84.6% and remains higher than the national average of 79.9% according to the Scientific Registry of Transplant Recipients 2011 annual report [11].

Our study helps to answer the clinical question of whether or not troponin elevation is due to the acute stressor of the recent transplantation or whether there is any potential cardiac-related mortality related to this elevation. Because of the many noncardiac causes of troponin elevation in this population, the dilemma exists as to whether or not obtaining a troponin level can be helpful in risk stratification in patients with acute cardiac changes following surgery. Interestingly, all of our patients within the intermediate TnT group were alive at followup, which suggests that slight elevations in troponin may, in fact, be associated with the recent surgery. Yet, patients with greater elevations were found to have a higher mortality rate, both cardiac and all-cause. The majority of those patients with the elevated TnT that deceased did so during a different hospitalization than the one in which the transplantation was performed. 

Our study found no statistical differences in risk factors, including history of coronary artery disease, history of myocardial infarction, and diabetes mellitus. There was a significant difference in incidence of hyperlipidemia and tobacco use. The elevated troponin group had a greater number of tobacco users, while the intermediate group had the most patients with hyperlipidemia. No patients in the elevated troponin group had hyperlipidemia. Such findings can be biased secondary to patients with higher risk factors having been denied transplantation and not included in the study, yet the presence or lack of risk factors should not be used to decide whether or not to pursue further cardiovascular testing with troponin elevations following transplantation.

This study showed that there was no difference in pre-transplantation cardiovascular testing, including echocardiogram and stress test results. It was expected that patients with increased rates of cardiovascular mortality would have had some type of baseline deficit on echocardiography or noninvasive stress testing prior to transplantation, but this was not the case. Our results suggest that the decision regarding cardiovascular work-up following transplant should not be solely based on the findings prior to transplant. 

Patients with elevated TnT were also noted to have higher overall MELD scores and INR values when compared with the other groups. There was a higher percentage of patients with renal dysfunction in the elevated TnT group compared with the others, yet this was not statistically different. This data suggest that perhaps those patients with elevated TnT levels are more acutely ill than the other groups and that the elevated in TnT is likely secondary to an alternative cause such as myopathy. Multiorgan system failure can also potentially cause TnT elevation, yet no difference in proportion of patients with multiorgan system failure was noted between the groups.

Electrocardiogram changes at the time of TnT elevation were not significantly different between the three groups. The negative and intermediate TnT groups did have a higher percentage of patients with normal electrocardiograms versus those in the elevated group which often had nonspecific ST-segment changes. While it is recommended that electrocardiogram changes not to be used solely to decide whether or not a patient following transplantation is at risk for cardiovascular complications, these findings do suggest that patients with an abnormal electrocardiogram in the setting of an elevated TnT may have worse outcomes.

Many limitations should be acknowledged, including the relatively small patient population. However, to our knowledge, this reflects the largest study to date evaluating the association between troponin and mortality following OLT. We acknowledge the many factors that may cause or contribute to an elevated troponin level and certainly there may be some caution to using this laboratory value to guide when to pursue invasive angiography, especially in the setting of recent transplantation. The lack of invasive testing adds merit to this assumption in that clinicians likely attributed the troponin elevation to surgery itself. The lack of invasive coronary assessment is a large boundary in this study as we cannot be certain as to the precise mechanism of troponin elevation. While these findings do point to patients in the elevated troponin group having worse liver disease and renal dysfunction, some may have been predisposed to perioperative infarction given their underlying risk factors. Patients in this study have at least two years of followup following transplantation, yet an additional limitation is the lack of longer-term followup. Patients in this study can also be deemed to be more acutely ill than patients who did not have TnT laboratory studies, yet the overall survival rate of our cohort is almost identical to that of our institution as a whole. 

## 5. Conclusion

 The significance of troponin elevation following transplantation is difficult to assess, especially considering the acuity of such patients. Our study shows that patients with elevated TnT levels are found to have a higher mortality rate than those with either normal or intermediate levels. Both negative and intermediate TnT levels are not associated with postoperative cardiovascular mortality in our cohort. Patients with elevated TnT levels do have a significantly higher rate of mortality, both all-cause and cardiac. Troponin evaluation following OLT may aid in the prognostic assessment of these patients. 

## Figures and Tables

**Table 1:** Baseline patient characteristics prior to transplantation.

	All patients *n* = 78	Troponin			*P* value
		Negative (≤0.01) *n* = 42	Intermediate (0.01–0.11) *n* = 28	Elevated (>0.11) *n* = 8	
Age at transplantation	58.2	56.1	61.7	60.5	*P* = 0.11
Sex					*P* = 0.078
Male	48 (61.5%)	30 (71.4%)	13 (46.4%)	5 (62.5%)	
Female	30 (38.5%)	12 (28.6%)	15 (53.6%)	3 (37.5%)	
History of coronary artery disease	12 (15.4%)	4 (9.5%)	6 (21.4%)	2 (25.0%)	*P* = 0.24
History of myocardial infarction	2 (2.6%)	1 (2.4%)	1 (3.6%)	0 (0.0%)	*P* = 1.00
Diabetes	25 (32.1%)	12 (28.6%)	11 (39.3%)	2 (25.0%)	*P* = 0.60
Hypertension	28 (35.9%)	16 (38.1%)	9 (32.1%)	3 (37.5%)	*P* = 0.79
Hyperlipidemia	13 (16.7%)	4 (9.5%)	9 (32.1%)	0 (0.0%)	*P* = 0.031
Tobacco use	29 (37.2%)	21 (50.0%)	4 (14.3%)	4 (50.0%)	*P* < 0.01
Echocardiogram (*n*)	75 (96.2%)	40 (95.2%)	27 (96.4%)	8 (100.0%)	
LVEF (%)	65.1	63.9	65.8	68.9	*P* = 0.10
RV mean pressure (mm Hg)	27.8	28.3	27.4	26	*P* = 0.62
LV diastolic size (mm)	48.5	49.6	46.7	47.9	*P* = 0.074
LV systolic size (mm)	30.2	31.3	28.9	28.1	*P* = 0.069
RV size					*P* = 0.45
Normal	72 (96.0%)	38 (95.0%)	27 (100.0%)	7 (87.5%)	
Mild enlargement	2 (2.7%)	1 (2.5%)	0 (0.0%)	1 (12.5%)	
Moderate enlargement	1 (1.3%)	1 (2.5%)	0 (0.0%)	0 (0.0%)	
Overall function					
Normal function	71 (94.7%)	38 (95.0%)	25 (92.6%)	8 (100.0%)	*P* = 0.47
Wall-motion abnormalities	4 (5.3%)	2 (5.0%)	2 (7.4%)	0 (0.0%)	
Pretransplantation stress test	71 (91.0%)	36 (85.7%)	28 (100.0%)	7 (87.5%)	*P* = 0.086

LVEF: left ventricular ejection fraction; RV: right ventricle; LV: left ventricle.

**Table 2:** Comparisons of types of liver disease.

	All patients	Troponin		
		Negative	Intermediate	Elevated
Primary liver disease (*n*)	78	42	28	8
Alcohol	10 (12.8%)	5 (11.9%)	2 (7.1%)	3 (37.5%)
Hepatitis C	30 (38.5%)	19 (45.2%)	9 (32.1%)	2 (25.0%)
NASH	11 (14.1%)	5 (11.9%)	4 (14.3%)	2 (25.0%)
PBC	5 (6.4%)	2 (4.8%)	3 (10.7%)	0 (0.0%)
PSC	5 (6.4%)	4 (9.5%)	1 (3.6%)	0 (0.0%)
Acetaminophen	1 (1.3%)	1 (2.4%)	0 (0.0%)	0 (0.0%)
Cryptogenic	13 (3.9%)	6 (14.3%)	6 (21.4%)	1 (12.5%)
Polycystic	2 (2.6%)	0 (0.0%)	2 (7.1%)	0 (0.0%)
Autoimmune	1 (1.3%)	0 (0.0%)	1 (3.6%)	0 (0.0%)
Secondary liver disease (*n*)	19 (24.4%)	13 (31.0%)	5 (17.9%)	1 (12.5%)
Alcohol	3 (15.8%)	2 (15.4%)	1 (20.0%)	0 (0.0%)
Hepatitis C	1 (5.3%)	0 (0.0%)	1 (20.0%)	0 (0.0%)
HC	15 (78.9%)	11 (8.5%)	3 (60.0%)	1 (100.0%)

NASH: nonalcoholic steatohepatitis; PBC: primary biliary cirrhosis; PSC: primary sclerosing cholangitis; acetaminophen: acetaminophen toxicity; HC: hepatocellular carcinoma.

**Table 3:** Laboratory data and electrocardiogram findings at time of troponin elevation.

	All patients	Troponin			*P* value
		Negative	Intermediate	Elevated	
Avg. troponin (ng/mL)	0.07	0.01	0.047	0.455	*P* < 0.001
MELD score	11.20	8.89	13.10	14.0	*P* = 0.003
INR	1.62	1.46	1.79	1.84	*P* = 0.011
Tbili (mg/dL)	4.18	4.16	4.18	3.75	*P* = 0.052
Cr (mg/dL)	1.14	0.095	1.25	1.73	*P* = 0.093
Renal dysfunction	28 (35.9%)	9 (21.4%)	13 (46.4%)	6 (75.0%)	*P* = 0.079
ECG changes					
None	40 (51.3%)	25 (59.5%)	14 (50.0%)	1 (12.5%)	
ST elevation	0 (0.0%)	0 (0.0%)	0 (0.0%)	0 (0.0%)	
ST depression	2 (2.6%)	0 (0.0%)	2 (7.1%)	0 (0.0%)	
Afib/flutter	8 (10.3%)	6 (14.3%)	1 (3.6%)	1 (12.5%)	
Nonspecific ST	17 (21.8%)	6 (14.3%)	7 (25.0%)	4 (50.0%)	
Sinus tachycardia	8 (10.3%)	4 (9.5%)	4 (14.3%)	0 (0.0%)	
New RBBB	2 (2.6%)	1 (2.4%)	0 (0.0%)	1 (12.5%)	
New LBBB	1 (1.3%)	0 (0.0%)	0 (0.0%)	1 (12.5%)	

MELD: model for endstage liver disease; INR: international normalized ratio; Tbili: total bilirubin; Cr: creatinine; ECG: electrocardiogram; Afib/flutter: atrial fibrillation/flutter; Nonspecific ST: nonspecific ST changes; RBBB: right bundle branch block; LBBB: left bundle branch block.

**Table 4:** Comparison of patient status following transplantation.

	All patients	Troponin			*P* value
		Negative	Intermediate	Elevated	
Age at followup	61.5	59.4	64.9	63.3	*P* = 0.06
Followup status					*P* = 0.001
Alive	68 (87.2%)	36 (85.7%)	28 (100.0%)	4 (50.0%)	
Deceased	10 (12.8%)	6 (14.3%)	0 (0.0%)	4 (50.0%)	
Cardiac	4 (40.0%)	1 (16.7%)	0 (0.0%)	3 (75.0%)	
Liver	1 (10.0%)	1 (16.7%)	0 (0.0%)	0 (0.0%)	
Sepsis	2 (20.0%)	2 (33.3%)	0 (0.0%)	0 (0.0%)	
Other	3 (30.0%)	2 (33.3%)	0 (0.0%)	1 (25.0%)	

## References

[1] Coss E, Watt KDS, Pedersen R, Dierkhising R, Heimbach JK, Charlton MR (2011). Predictors of cardiovascular events after liver transplantation: a role for pretransplant serum troponin levels. *Liver Transplantation*.

[2] Plotkin JS, Scott VL, Pinna A, Dobsch BP, De Wolf AM, Kang Y (1996). Morbidity and mortality in patients with coronary artery disease undergoing orthotopic liver transplantation. *Liver Transplantation and Surgery*.

[3] Watt KDS, Pedersen RA, Kremers WK, Heimbach JK, Charlton MR (2010). Evolution of causes and risk factors for mortality post-liver transplant: results of the NIDDK long-term follow-up study. *American Journal of Transplantation*.

[4] Parekh NK, Hynan LS, De Lemos J (2007). Elevated troponin I levels in acute liver failure: is myocardial injury an integral part of acute liver failure?. *Hepatology*.

[5] Pateron D, Beyne P, Laperche T (1999). Elevated circulating cardiac troponin I in patients with cirrhosis. *Hepatology*.

[6] Keeffe BG, Valantine H, Keeffe EB (2001). Detection and treatment of coronary artery disease in liver transplant candidates. *Liver Transplantation*.

[7] Keller T, Zeller T, Peetz D (2009). Sensitive troponin I assay in early diagnosis of acute myocardial infarction. *New England Journal of Medicine*.

[8] Adams JE, Bodor GS, Davila-Roman VG (1993). Cardiac troponin I: a marker with high specificity for cardiac injury. *Circulation*.

[9] Ferguson JL, Beckett GJ, Stoddart M, Walker SW, Fox KAA (2002). Myocardial infarction redefined: the new ACC/ESC definition, based on cardiac troponin, increases the apparent incidence of infarction. *Heart*.

[10] Møller S, Henriksen JH (2002). Cirrhotic cardiomyopathy: a pathophysiological review of circulatory dysfunction in liver disease. *Heart*.

[11] (2012). Liver Transplant. http://www.mayoclinic.org/liver-transplant/liver-fl-survival-graph.html.

